# Is There a Role for SARS-CoV-2/COVID-19 on the Female Reproductive System?

**DOI:** 10.3389/fphys.2022.845156

**Published:** 2022-03-02

**Authors:** Silvia D’Ippolito, Francesca Turchiano, Amerigo Vitagliano, Gennaro Scutiero, Antonio Lanzone, Giovanni Scambia, Pantaleo Greco

**Affiliations:** ^1^Dipartimento di Scienze della Salute della Donna, del Bambino e di Sanità Pubblica, Fondazione Policlinico Universitario A. Gemelli Istituto di Ricovero e Cura a Carattere Scientifico (I.R.C.C.S.), Rome, Italy; ^2^Dipartimento di Scienze Mediche, Università degli studi di Ferrara, Ferrara, Italy; ^3^Dipartimento di Scienze della Vita e Sanità Pubblica, Università Cattolica del Sacro Cuore, Rome, Italy

**Keywords:** Coronavirus disease (COVID-19), SARS-CoV-2, female fertility, pregnancy, SARS-CoV-2 colonization

## Abstract

Coronavirus disease (COVID-19) has emerged as a very serious pandemic caused by the rapidly evolving transmission of the coronavirus SARS-CoV-2. Since its outbreak in 2020, the SARS CoV-2 has represented an important challenge for the physicians due to its well known respiratory sequelae. To date, the role of SARS-CoV-2 infection on organs and systems other than lungs and respiratory tract remains less clear. In particular, it remains to be investigated whether the reproductive system can be affected by the SARS-CoV-2 in the long term-period or, in alternative, drugs used to treat COVID-19 might impact the reproductive systems and, in turn, fertility. What is known is that SARS-Cov-2 binds to target cells of host through different receptors including angiotensin-converting enzyme 2 (ACE2), neuropilin-1, AXL and antibody-FcɣR complexes. ACE2 physiologically regulates both the expression of angiotensin II (Ang II) as well as Ang-(1-7) to exerts its physiological functions. The reproductive system abundantly expresses ACE2 and produces Ang-(1-7), starting from precursors which are locally generated or derived from systemic circulation. Ang-(1-7) plays an important role of stimulus to the growth and maturation of ovarian follicle as well as to ovulation. Also human endometrium expresses Ang-(1-7), mainly during the post-ovulatory phase. Animal and human observational studies demonstrated that Ang-(1-7) is involved in the maternal immune response to pregnancy and its deficiency is associated with a defective placenta development. In our manuscript, we review the current knowledge about whether SARS-CoV-2 may impact the female reproductive system. We further explain the possible molecular mechanism by which SARS-CoV-2 might affect ovarian, endometrial and female genital tract cells.

## Introduction

Coronavirus disease (COVID-19) represents the well known emerging infectious disease caused by the widespread transmission of the Severe Acute Respiratory Syndrome Coronavirus-2 (SARS-CoV-2) (Atzrodt et al., 2020). Since its outbreak in 2020, the SARS CoV-2 has represented an important challenge for the physicians due to its important sequelae. A large amount of literature describes the classical symptoms of this infectious disease, mainly related to airway involvement, including dry cough, dyspnea, fever. Less common symptoms include tiredness, musculoskeletal pain, headache, gastrointestinal disorders, together with smell and taste loss (Hu et al., 2021).

Increasing research are now reporting the consequences of the infection from SARS-CoV-2 on systems other than respiratory system (Fu et al., 2020; Madjid et al., 2020). In particular, the investigation whether the reproductive system can be affected by the coronavirus is in progress (Selek et al., 2021). Our knowledge about the viral infection sequelae on the reproductive system is continuously updated. Of interest, in spite of a similar infection rate in both sexes, several studies reported the sex disparity in terms of COVID-19 severity and outcome. Differences in the processes of infection, immune reaction to the virus and in the progression of the cascade of inflammation have been advocated to explain the observed disparity (Raza et al., 2021; Traish, 2021). In our review we will evaluate whether SARS-Cov-2 might play a role on the female reproductive system. It is important to consider the effects of the viral infection on the reproductive system, in order to provide an updated approach for reproductive age women.

## Severe Acute Respiratory Syndrome Coronavirus-2 and Receptor

**FIGURE 1:**
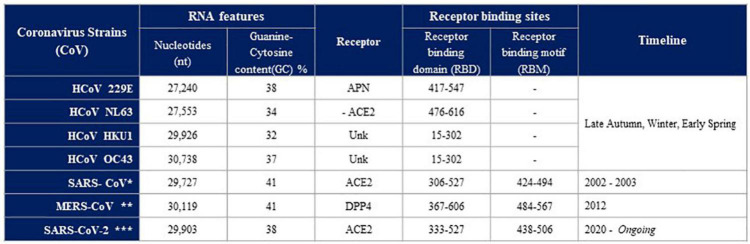
Human coronaviruses (HCoVs) comparison of RNA Features, Receptor, Receptor binding sites, Timeline. To date, seven human coronaviruses (HCoVs) have been detected: HCoV-229E, HCoV-NL63, HCoV-HKU1, HCoV-OC43, severe acute respiratory syndrome coronavirus (SARS-CoV*), Middle East respiratory syndrome coronavirus (MERS-CoV**) and SARS-CoV-2***. Four of these viruses, including HCoV-229E, -NL63, -HKU1, and -OC43, usually cause mild-to-moderate respiratory diseases with a seasonal pattern. Three new HCoVs have recently emerged with a significant mortality rate. Despite the fact that all HCoVs share similarities in viral replication, they differ in their accessory proteins, incubation period and pathogenicity (Modified by Kesheh et al., 2021).

**FIGURE 2:**
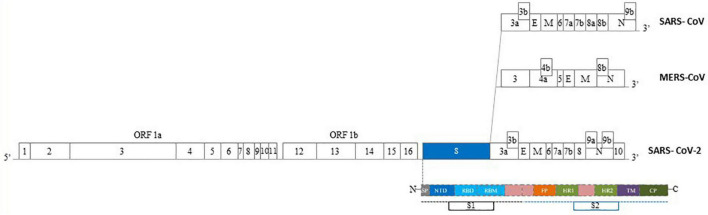
Epidemics Human Coronaviruses (HCoVs) structure of genome. The genomes of epidemics Human CoVs contain a single-stranded, positive-sense RNA ranging of 27–32 kb in size. One third of the genome, expressed at the 3′ terminal, encodes the main proteins of the virus involved in the virus-cell receptor binding and virion activity. In particular, four structural proteins: E: envelope protein, M: membrane protein, N: nucleocapsid protein, S: spike protein. The S protein of HCoVs consists of two subunits, S1 and S2. S1 subunit consist of a C-Terminal Domain (CTD) – also named Receptor Binding Domain (RBD) – and a N-Terminal Domain (NTD). S2 subunit contains a fusion peptide (FP), transmembrane domain (TM) region, heptad repeat 1-2 (HR1, HR2). SP, signal peptide; CP, cytoplasmic peptide; ORF, open reading frame (Modified by Zhao et al., 2020b).

Coronaviruses (CoVs) are viruses with single-stranded positive-sense RNA. They are named for their surface showing crownlike spikes and show a rapid ability of mutation (Figure 1). Their subfamily, called Coronavirinae, is further classified into four groups, two of them (α and β) are involved in human respiratory or intestinal infections (Cheng et al., 2007). Likewise SARS-CoV and MERS-CoV, SARS-CoV-2 belongs to the β-CoV type (Figure 2). Its structure is based on four major structural proteins: the nucleocapsid, the spike protein, the membrane and the envelope protein (Zhang et al., 2021). The nucleocapsid is complexed with the viral RNA to create a helical capsid. The spike protein together with the envelope and membrane form the membrane proteins of Sar-CoV-2 (Lee et al., 2020; Zhang et al., 2021). The spike protein is a glycoprotein forming superficial peplomers through which the virus binds to angiotensin-converting enzyme 2 (ACE2). ACE2 is a membrane-bound aminopeptidase which acts as the main receptor for SARS-CoV-2. To date, ACE2 has been recognized as the crucial agent regulation the system of renin-angiotensin system (RAS). This system lead to the conversion of the angiotensin II (Ang II) to the angiotensin 1-7 (Ang[1-7]), both hormones with opposing activities. Beyond vasoconstriction, Ang II mediates pro-inflammatory, pro-fibrotic, pro-apoptotic and tissue remodeling properties. On the contrary, Ang-(1-7) shows anti-inflammatory and anti-fibrotic properties (Bourgonje et al., 2020; Lee et al., 2021). Infection with SARS-CoV-2 leads to an impaired activity and expression of ACE2. As a consequence, increased levels of circulating Ang II are observed in infected patients hence leading to the enormous inflammatory and fibrotic transformation observed in the lungs from positive individuals (Kuba et al., 2005; Verdecchia et al., 2020).

The ACE2 is predominantly expressed on airways, intestine, kidney, heart, endothelium as well as on hematopoietic and immune cells (Ackermann et al., 2020; García, 2020; Hoffmann et al., 2020; Sungnak et al., 2020a; Wiersinga et al., 2020). This broad expression implies a wide range of effects in the host systems. Also ACE2 shows a variety in the efficiency in each system indicating a varied susceptibility to the viral infection.

To favor its entrance into the host cells, SARS-CoV-2 takes advantage of some surface proteases. One of the most studied is the transmembrane protease serine 2 (TMPRSS2) expressed on the cell surface of human pneumocytes, intestinal enterocytes, kidney cells, and endothelial cells. TMPRSS2 is involved in the cleavage of the viral spike protein. After the cleavage, the resulting conformational change allows the permanent fusion of the virus to the host cell membranes (Stanley et al., 2020; Sungnak et al., 2020b). Importantly, the coexpression of TMPRSS2 and ACE2 can be observed not only in the respiratory system, mainly the lung, but also in the intestine and kidney and the vascular system. This localization explains why SARS CoV-2 mostly damages these organs (Ackermann et al., 2020; García, 2020; Hoffmann et al., 2020; Sungnak et al., 2020a,b; Wiersinga et al., 2020; Pivonello et al., 2021). Also the reproductive systems coexpresses ACE2 and TMPRSS2 genes, in particular in ovarian, endometrial placental and testicular cells with different degree of expression (Qi et al., 2020; Sungnak et al., 2020a,b).

Further receptors possibly modulating the viral entry into the target cells include receptor basigin (BSG/CD147) and the cysteine protease cathepsin L (CTSL) (Stanley et al., 2020).

## The Impact of Severe Acute Respiratory Syndrome Coronavirus-2 on the Female Reproductive System

The components of RAS, including the ACE2, are expressed in the female reproductive system, mostly in the ovary. This makes them potential targets for SARS-CoV-2 activity. Angiotensin II is mainly expressed in granulosa cells. Angiotensin-(1-7) is expressed in theca-interstitial cells. Both are involved in the processes of oocyte maturation up to the corpus luteum development, in the regulation of follicular involution as well as in the processes of ovarian hormonal production (Herr et al., 2013; Pan et al., 2013; Yan et al., 2020). The ACE2 and Ang-(1-7) are found in all stages of follicular development suggesting a role in fertility (Pan et al., 2013; Yan et al., 2020; Lee et al., 2021). Actually, Ang-(1-7) can be collected from follicular fluid during ovarian stimulation techniques and their levels positively correlate with the proportion of mature oocytes, indicating that Ang-(1-7) might represent an indirect marker of human oocyte maturation (Cavallo et al., 2017). SARS-CoV-2 downregulates the ACE2, this leads to increased levels of Ang II, whose proinflammatory, profibrotic and proapoptotic activities have been reported. As a consequence, this might impact ovarian functions and induce a state of increased ovarian oxidative stress. Recently, Stanley et al. (2020) aimed at identifying cell types in the female ovaries coexpressing both the ACE2 and TMPRSS2. No coexpression was found in ovarian somatic cells. RNA expression of TMPRSS2 in 18 samples of human cumulus cells was shown to be low or absent (Stanley et al., 2020). These observations might explain the reason why SARS-CoV-2 difficultly is found in the ovarian tissue.

In the endometrium, stromal and epithelial cells also express RAS (Vinson et al., 1997; Herr et al., 2013; Yan et al., 2020) with fluctuation during the cycle. ACE2 and Ang-(1-7) expression is predominant in the secretory phase. The RAS system is involved in maintaining regular menstrual cycles, facilitating regeneration of blood vessels and initiating menstruation. These processes are strictly regulated by the balanced expression of Ang II and Ang-(1-7). In particular, Ang II represents a stimulatory factor whereas Ang-(1-7) an inhibitory one (Yan et al., 2020). Given the ACE2 expression, it would be expected that the process of endometrial regeneration, proliferation and, consequently, implantation, might be affected by SARS-CoV2 infection. However, to our knowledge, there are no studies demonstrating an endometrial susceptibility to the virus (Henarejos-Castillo et al., 2020). Nevertheless, since ACE2 endometrial localization has been positively correlated with age, it has been suggested an age-related endometrial predisposition to viral infection (Abhari and Kawwass, 2020; Markiewicz-Gospodarek et al., 2021). Of interest, SARS-Cov-2 encodes proteins able to activate the assembly of inflammasome NLRP3 (Man et al., 2017; Lee et al., 2020). Inflammasome, important component of the innate immunity, represents one of the first defenses against viral infections. Among the agents able to activate inflammasome, also viral RNA are included. Once triggered, NLRP3 recruits Caspase-1, which, in turn, enhances the expression of interleukin (IL) -1β and -18, respectively (Sandall et al., 2020; Zhao et al., 2020a). It has been previously reported an increased unfavorable expression of both NLRP3 and proinflammatory cytokines in the endometrium from women with history of recurrent miscarriages (D’Ippolito et al., 2016). Whether SARS-CoV-2 infection might activate NLRP3 and induce an endometrial disfunction needs to be further investigated.

Vagina and cervix lack of ACE2 expression. Consistently Cui et al. (2020) tried to assess the presence of SARS-CoV-2 in these tissues. To this end, they collected vaginal fluid and cervical exfoliated cells from 35 both reproductive-age and post-menopausal women with mild to moderate SARS-CoV-2 disease (Cui et al., 2020). They did not found SARS-CoV-2 in the lower genital tract. In this direction, two further studies have been set including vaginal fluid and cervical exfoliated cells collected from 12 COVID-19 hospitalized pregnant women and 10 women with severe COVID-19 pneumonia admitted to an Intensive Care Unit, respectively. All the analyzed samples were negative for SARS-CoV-2 (Aslan et al., 2020; Qiu et al., 2020). Consistently, a systematic review on pregnant women confirmed the absence of the virus in the vaginal samples from all tested women (Juan et al., 2020). In contrast, Shwartz et al. analyzed premenopausal and postmenopausal women during acute viral infection. They found a vaginal RT-PCR positivity of SARS-CoV-2 in 2/35 cases (5.7%; one pre-menopausal and the other one post-menopausal; Schwartz et al., 2021). Also a previous case report the vaginal viral positivity in a 23-year-old primiparous patient (Vivanti et al., 2020). We are not able to explain such discrepancies. The small number of patients, as well as the heterogeneity in the age of included women and varying degrees of disease severity could contribute to the contrasting results. Furthermore, whether a correlation exists between the viral load and/or viremia and the possibility to detect the virus at vaginal levels should be further assessed. In this direction, an adequate counseling to a SARS-CoV-2 positive woman at birth should include the information about a possible vaginal colonization during vaginal delivery.

Taken together, these studies suggest the female reproductive system by expressing the ACE2 shows a great potential to be susceptible to SARS-CoV-2 entry. In spite of this, no known damage to the female reproductive system has been reported. We are not able to completely explain such findings. The potential impact of the infection on female fertility needs to be adequately assessed mainly in the long term follow-up.

## Severe Acute Respiratory Syndrome Coronavirus-2 and Placenta

### Implications for Pregnancy Outcome

The elements of the Renin Angiotensin System (RAS) have been identified early (6 weeks of gestation) in the placenta (Levy et al., 2008; Dashraath et al., 2020). In particular, their expression has been reported in the maternal decidua and spiral arteries, as well as in cytotrophoblasts, syncytiotrophoblasts, fetal capillaries in primary and secondary villi. Also, in the umbilical cord, ACE2 is localized in smooth muscles and the vascular endothelium (Levy et al., 2008; Dashraath et al., 2020). The function of RAS in the placenta is not well known. It has been suggested that an altered placental RAS expression might contribute to defective placentation, hence leading to pre-eclampsia or intrauterine growth restriction (Ghadhanfar et al., 2017; Lumbers et al., 2019). This might represent a mechanism by which placental COVID-19 infection may impact pregnancy outcome. The ACE2 placental expression suggests that placental tissue, at different levels, may represent the potential risk for SARS-CoV-2 placental infection. According to the literature, infected women, especially in the presence of pneumonia, appear to have an increased risk of preterm birth (before 37 weeks of gestation), mainly iatrogenic, and cesarean delivery, likely related to severe maternal illness. To date, evidence confirming the SARS-CoV-2 cross through the placenta and ability to infect the fetus (transplacental COVID-19 infection) is heterogeneous (Schwartz, 2020b; Schwartz and Morotti, 2020a; Vivanti et al., 2020; Zeng et al., 2020). In spite of placental detection of the virus, the neonates remain negative during their early days of life (Edlow et al., 2020). Possible contamination may occur soon after birth through maternal blood, vaginal secretions and anorectum route (Carosso et al., 2020).

During early phase of COVID 19 pandemic, initial reports regarding the infection and pregnancy included a limited number of pregnant women. To overcome this limitation, the authors analyzed the pregnancy outcome by making a comparison among the most important coronavirus epidemics in the recent past, in particular COVID-19, MERS and SARS. Dashraath et al. (2020) reported the obstetric outcome of 84 pregnant women: 55 pregnancies (65.4%) affected by SARS-CoV-2, 12 (14.3%) by MERS, and 17 (20.3%) by SARS. When considering COVID-19 they reported a rate of miscarriage/stillbirth, intrauterine restriction and preterm birth of 2, 9, and 43% respectively. In MERS infected women the rate of miscarriage/stillbirth, intrauterine restriction and preterm birth was 18, 9, and 27%, respectively. Finally, SARS infected women registered a miscarriage/stillbirth rate of 25%, intrauterine growth restriction rate of 13%, and a preterm birth of 25% (Dashraath et al., 2020).

Also Di Mascio et al. (2020) analyzed the obstetric outcome in 79 women affected by CoV infections including SARS-CoV-2, MERS and SARS. For all CoV infections, the following obstetric complications were found: pregnancy loss (39.1%), preterm birth < 37 weeks (24.3%), premature pre-labor rupture of membranes (20.7%), preeclampsia (16.2%), and fetal growth restriction (11.7%). The rate of cesarean section and perinatal death was 84 and 11.1%, respectively. About 57% of newborns were admitted to neonatal intensive care unit (NICU). When considering SARS-CoV-2 infected pregnancies, premature birth (<37 weeks) occurring in 41.1% of cases was the predominat pregnancy complication. Perinatal death occurred in about 7% of cases. No cases of vertical transmission were registered (Di Mascio et al., 2020). The main limitation of the above reported studies is that they report the summary of all CoVs-related illnesses and collect data from a relative limited number of pregnancies. A most recent systematic review, by Khalil et al. (2020) analyzed 17 studies on a total of 2,567 pregnant women affected by SARS-CoV-2. The authors found as most common obstetric complication the iatrogenic preterm birth. Generally the preterm birth was not indicated by fetal distress, rather by COVID-19 sequelae including sever maternal pneumonia, fear of sudden maternal decompensation. Cases of maternal mortality occurred rarely (less than 1%). Twelve stillbirths and four neonatal deaths were recorded. Rare cases of neonatal SARS-CoV-2 PCR positivity have been found (about 1.5%), suggesting a probable vertical transmission (Khalil et al., 2020).

In line with this analysis, Allotey et al. (2020) also found a significantly higher rate of preterm birth and of neonatal NICU admission in COVID-19 pregnant women as compared to negative pregnant women.

Altogether, this evidence suggest the difficulty to demonstrate a direct effect of SARS-COV-2 on placental tissue and consequently on pregnancy outcome. The demonstration that the most frequent obstetric complication is iatrogenic preterm birth confirm that pregnancy is affected by SARS-CoV-2 indirectly, through its important sequelae on the maternal system. Several important limitations should be considered during the analysis of the results. In particular, it has been reported that the most significant percentage of pregnant women (up to 90%) who are infected with SARS-CoV-2 remains asymptomatic (Khalil et al., 2020). Therefore, in the absence of an universal testing for SARS-CoV-2 the impact of the virus on pregnancy outcome could result underestimated. Also, during pandemic the maternal care services worldwide have changed and we are not able to evaluate possible indirect effects of COVID on pregnancy outcome. Also, a significant proportion of pregnancies affected by COVID-19 are detected near the delivery date, whereas data related to early pregnancy viral exposure are difficultly collected.

## Author Contributions

SD’I and PG: conceptualization, methodology, and writing original draft. SD’I and FT: formal analysis of the scientific literature. FT: figure preparation. SD’I, FT, AV, GeS, AL, GiS, and PG: review and editing of the original draft. All authors contributed to the article and approved the submitted version.

## Conflict of Interest

The authors declare that the research was conducted in the absence of any commercial or financial relationships that could be construed as a potential conflict of interest.

## Publisher’s Note

All claims expressed in this article are solely those of the authors and do not necessarily represent those of their affiliated organizations, or those of the publisher, the editors and the reviewers. Any product that may be evaluated in this article, or claim that may be made by its manufacturer, is not guaranteed or endorsed by the publisher.
